# Clodronate Liposomes Improve Metabolic Profile and Reduce Visceral Adipose Macrophage Content in Diet-Induced Obese Mice

**DOI:** 10.1371/journal.pone.0024358

**Published:** 2011-09-12

**Authors:** Bin Feng, Ping Jiao, Yaohui Nie, Thomas Kim, Dale Jun, Nico van Rooijen, Zaiqing Yang, Haiyan Xu

**Affiliations:** 1 Key Laboratory of Agricultural Animal Genetics, Breeding and Reproduction of Ministry of Education, College of Life Science and Technology, Huazhong Agricultural University, Wuhan, China; 2 Department of Molecular Cell Biology, Faculty of Medicine, Vrije Universiteit, Amsterdam, The Netherlands; 3 Alpert Medical School, Hallett Center for Diabetes and Endocrinology, Brown University, Providence, Rhode Island, United States of America; Université Paris Descartes, France

## Abstract

**Background:**

Obesity-related adipose inflammation has been thought to be a causal factor for the development of insulin resistance and type 2 diabetes. Infiltrated macrophages in adipose tissue of obese animals and humans are an important source for inflammatory cytokines. Clodronate liposomes can ablate macrophages by inducing apoptosis. In this study, we aim to determine whether peritoneal injection of clodronate liposomes has any beneficial effect on systemic glucose homeostasis/insulin sensitivity and whether macrophage content in visceral adipose tissue will be reduced in diet-induced obese (DIO) mice.

**Methodology/Principal Findings:**

Clodronate liposomes were used to deplete macrophages in lean and DIO mice. Macrophage content in visceral adipose tissue, metabolic parameters, glucose and insulin tolerance, adipose and liver histology, adipokine and cytokine production were examined. Hyperinsulinemic-euglycemic clamp study was also performed to assess systemic insulin sensitivity. Peritoneal injection of clodronate liposomes significantly reduced blood glucose and insulin levels in DIO mice. Systemic glucose tolerance and insulin sensitivity were mildly improved in both lean and DIO mice treated with clodronate liposomes by intraperitoneal (ip) injection. Hepatosteatosis was dramatically alleviated and suppression of hepatic glucose output was markedly increased in DIO mice treated with clodronate liposomes. Macrophage content in visceral adipose tissue of DIO mice was effectively decreased without affecting subcutaneous adipose tissue. Interestingly, levels of insulin sensitizing hormone adiponectin, including the high molecular weight form, were significantly elevated in circulation.

**Conclusions/Significance:**

Intraperitoneal injection of clodronate liposomes reduces visceral adipose tissue macrophages, improves systemic glucose homeostasis and insulin sensitivity in DIO mice, which can be partially attributable to increased adiponectin levels.

## Introduction

Obesity has become a huge public health burden since it is related to the development of many chronic diseases such as type 2 diabetes. Elevated inflammatory markers in circulation have been commonly observed in obese and diabetic patients [1]–[5]. Inflamed adipose tissue and circulating mononuclear cells are possible sources for releasing increased inflammatory markers [6], [7]. Recently, extensive studies have been performed to determine the role of adipose inflammation in development of obesity-related insulin resistance and type 2 diabetes [8]–[11]. Animal models have shown strong evidence that adipose tissue originated low degree inflammation may play a role for inducing systemic insulin resistance in obese state. Multiple types of immune cells have been identified in obese adipose tissue, such as macrophages, neutrophils, T cells and mast cells [6], [12]–[16]. Macrophages accumulated and activated in adipose tissue in obesity have been shown to secrete a variety of proinflammatory cytokines, which potentially contribute to obesity-related chronic inflammation [6], [17]. Polarization of macrophages from anti-inflammatory M2 state to proinflammatory M1 state is thought to be the mechanism of macrophage activation induced by high fat diet [18]. Diet induced obese mice with reduced adipose macrophage infiltration have improved insulin sensitivity, as observed in mice deficient in CCR2, osteopontin and CXCL14 [19]–[21]. Proinflammatory macrophages accumulated in obese adipose tissue express CD11c and conditional depletion of CD11c positive cells through genetic approach also restores insulin sensitivity [22]. Consistently, mice with increased adipose tissue macrophage accumulation have compromised insulin sensitivity, as shown in *cbl-b* deficient and MCP-1 transgenic mice [23]–[25].

Ablation of adipose tissue macrophage content will be important to evaluate the therapeutic feasibility for improving obesity-related insulin resistance. The genetic approach that was used to deplete adipose tissue CD11c+ macrophages implied bone marrow transplant to introduce CD11c-diphtheria toxin receptor (DTR) bone marrow into lethally irradiated wild type mice and followed by injection of diphtheria toxin. This approach not only depletes CD11c+macrophages in adipose tissue, it is also expected to deplete macrophages in other tissues such as lung [26]. In addition, CD11c is not a macrophage specific marker, transgenic expression of DTR-EGFP under a cd11c promoter also resulted in deletion of dendritic cells, activated CD8+ T cells and plasma B cells [26]. Clodronate liposomes have been widely used to specifically ablate macrophages in many tissues through inducing macrophage apoptosis. Intravenous injection of clodronate liposomes is sufficient for depletion of macrophages in liver and spleen but was insufficient for reaching other organ/tissue in peritoneal cavity [27], [28]. Clodronate liposomes can not penetrate vascular barriers so local injections have been applied for eliminating macrophages in most of the tissues such as pancreas, lung, lymph nodes, testis, joints and central nervous system. We successfully reduced macrophage content in visceral adipose tissue of DIO mice by intraperitoneal injection (i.p.) of clodronate liposomes. Two previous studies applied a small amount of clodronate liposomes to DIO mice (approximately 4–5 mg/kg) which was not expected to have an effect on visceral adipose macrophage content and conflicting results were reported on hepatosteatosis [29], [30]. A third report with unspecified amount of clodronate liposomes administered into DIO mice did not investigate the effect on glucose homeostasis/systemic insulin sensitivity or macrophage content in adipose tissue [31]. Therefore, our work provides novel information regarding the beneficial effect of clodronate liposomes on glucose homeostasis which is associated with reduction of macrophage content in visceral but not subcutaneous adipose tissue [30], [31].

## Methods

### Ethics statement

Animal experiments were approved by the Institutional Animal Care and Use Committee of Rhode Island Hospital (protocol 0293-07 and de novo renew 0161-10).

### Reagents

Clodronate liposomes were purchased from clodronateliposomes.org (Vrije Universiteit, Netherlands) at the concentration of 1 mg/ml and prepared as previously described [32]. Clodronate was a gift of Roche Diagnostics GmbH, Mannheim, Germany. Adiponectin ELISA kits were purchased from R&D Systems. High molecular weight mouse adiponectin ELISA kit was purchased from BioVendor. IL-6 and TNFα ELISA kits were purchased from BioLegend. Leptin ELISA kit was purchased from RayBiotech. F4/80 antibody was purchased from Serotec. ATP, horse radish peroxidase, glycerol phosphate oxidase, glycerol kinase and lipase were purchased from Sigma. Amplex red was purchased from Molecular Probes. Humulin R was purchased from Eli Lilly Inc.

### Histology

Epididymal, mesenteric, peri-nephric adipose tissue, and liver were harvested from mice and weighted. For adipose tissue, one portion was used to isolate primary adipocytes and stromal vascular cells; one portion was frozen in liquid nitrogen immediately and stored at −80°C; and one portion was fixed in 10% formalin for one day, then transferred to 70% ethanol and paraffin embedded. For liver, one portion was fixed in 10% formalin for histology and the rest was frozen in liquid nitrogen immediately and stored at −80°C. Hematoxylin-eosin staining and F4/80 immunohistochemistry were done in Rhode Island Hospital core laboratory. F4/80 antibody was used at a 1∶50 dilution. Slides were counterstained with methyl green. To count macrophages, slides were viewed with a Nikon E800 microscope, using a 40x PlanApo objective. Cells stained for F4/80 were visually counted and recorded. Eight randomly selected views were counted per slide.

### Isolation of primary adipocytes and stromal vascular cells

Epididymal and mesenteric fat pads from DIO mice were excised, weighted, and rinsed in isolation buffer. Fat pads were then cut into small pieces in isolation buffer. Type I collegenase was added at a final concentration of 1 mg/ml. Minced fat pads were digested at 37°C in a shaking water bath at 100 rpm for 30 minutes. Digested tissues were then filtered through a metal mesh with a pore size of 380 µM to get single cell suspension. Cell suspensions were centrifuged at 1000 rpm for 3 min at room temperature. Floating adipocytes were transferred to clean tubes and rinsed twice with isolation buffer. The pellets, which contain stromal vascular cells, were resuspended in red blood cell lysis buffer to remove red blood cells. Next, stromal vascular cells were rinsed in PBS and filtered through a metal mesh with a pore size of 94 µM to get single cells.

### Tissue triglyceride measurement

Tissues were weighed and homogenized for 40 seconds in 30 volumes of ethanol. Samples were then vortexed for 5 minutes and settled for 5 minutes at room remperature. One milliliter of supernatant was transferred to a clean 1.5 ml tube and centrifuged at 15,000 g for 10 minutes at room temperature. For measuring triglyceride, five microliters of triglyceride standards or samples were mixed with one hundred microliters of reaction buffer (100 mM Tris, pH 7.4, 1 mM MgCl_2_ 0.05 mM ATP, 0.2 U/ml horse radish peroxidase, 1 U/ml glycerol phosphate oxidase, 2 U/ml glycerol kinase, 25 U/ml lipase, and 0.05 mM amplex red) and incubated for thirty minutes at 37°C. The fluorescence was read at excitation 530 nM/emission 590 nM using Synergy 4 plate reader (BioTek Instruments, Inc.).

### RNA extraction and real-time PCR analysis

RNA samples were extracted from tissues or isolated cells using the TRIzol reagent (Invitrogen, Carlsbad, CA) or the RNAeasy kit (Qiagen, Valencia, CA) according to the manufacturer's instructions. DNase I-treated RNA samples were reverse-transcribed with SuperScript III reverse transcriptase (Applied Biosystems, Carlsbad, CA) and random hexamers (Invitrogen, Carlsbad, CA) to generate cDNA. Real-time PCR analysis was performed using Power SYBR Green RT-PCR Reagent (Applied Biosystems, Carlsbad, CA) on ABI Prism thermal cycler model StepOnePlus (Applied Biosystems, Carlsbad, CA). Each 15-µl PCR reactions contained 1× reaction mix, 5.5 mM MgSO4, 300 nM forward primer, 300 nM reverse primer. The thermal cycling program was 50°C for 2 minutes, followed by 95°C for 10 minutes for 1 cycle, then 95°C for 15 seconds, followed by 60°C for 1 minute for 40 cycles. Melting curve analysis was performed to ensure the specificity of primers. Beta-actin was used as a reference gene in each reaction.

### Mouse models

Male C57BL/6J mice were purchased from the Jackson Laboratory at the age of 3 weeks. After one week of acclimation, mice were fed on either a normal chow diet (5% kcal from fat) or a high-fat diet (60% kcal from fat, D12492, Research Diets). Mice were injected with approximately 110 mg/kg of clodronate liposomes i.p. or equal volume of PBS liposomes. The injection was repeated three days later and experiments were performed 6–7 days after the first injection. For insulin tolerance tests, 22.1-week old DIO mice with 18.1-week on high fat diet and 25-week old lean mice were used for injection of clodronate liposomes. Mice were fasted for 6 hours and injected with insulin at the dose of 1 U/kg for DIO mice and 0.5 U/kg for lean mice. Blood glucose levels were measured using the AlphaTRAK blood glucometer (Abbott Animal Health) every 15 minutes up to 90 minutes. For glucose tolerance tests, 22.4-week old DIO mice with 18.4-week on high fat diet and 25-week old lean mice were used for injection of clodronate liposomes. Mice were fasted overnight and then injected with glucose at the dose of 1 g/kg for DIO mice and 2 g/kg for lean mice. Blood glucose levels were measured every 15 minutes up to 90 minutes. Tissues were harvested 1–2 days after GTT experiments. For insulin signaling studies, 24.6-week old DIO mice with 20.6-week on high fat diet were used for injection of clodronate liposomes. Insulin was injected into DIO mice through portal vein at the dose of 0.75 U/kg. Five minutes post insulin injection, tissues were collected and immediately frozen in liquid nitrogen. For intravenous injection of clodronate liposomes, 22.5 week old DIO mice with 18.5-week on high fat diet were used.

### Hyperinsulinemic-euglycemic clamp study

Mice were cannulated through jugular vein seven to ten days prior to experiments. DIO mice fed on a high fat diet for 25–29.5 weeks were used for injection of clodronate liposomes post surgery. The day before experiments, mice were fasted overnight. Next day mice were infused with HPLC purified D-[3-^3^H]-glucose (Perkin Elmer) at the rate of 0.05 µCi/min during a 2 h basal period. Blood glucose levels were measured at 60 and 120 minutes and blood samples were collected at the end of basal period. After the basal period, a bolus of human insulin (Humulin R) was infused for 3 min (16 mU/kg), which equals to the entire amount of insulin used in a two hour clamp. A 120-min hyperinsulinemic-euglycemic clamp was then performed through infusion of D-[3-^3^H]-glucose at the rate of 0.1 µCi/min plus continuous infusion of human insulin at the rate of 2.5 mU/kg/min. A solution of 25% dextrose was infused at variable rates to maintain blood glucose concentrations at basal levels. Infusions were performed using microdialysis pump (CMA/Microdialysis). Blood glucose levels were measured every 10 minutes. To estimate insulin-stimulated glucose uptake in individual tissues, 2-deoxy-D-[1-^14^C]-glucose (Perkin Elmer) was administered as a bolus of 10 µCi 75 minutes after the start of clamp. Blood samples were taken at 80, 85, 90, 100, 110, and 120 minutes during the clamp period for determining plasma concentrations of ^3^H-glucose, ^3^H_2_O, and 2-deoxy-D-[1-^14^C]-glucose. At the end of clamp, mice were sacrificed for collecting gastronemius muscles and epididymal fat tissue. Each tissue was frozen immediately in liquid nitrogen and stored at −80°C for further analysis. For determination of plasma concentrations of ^3^H-glucose and 2-deoxy-D-[1-^14^C]-glucose, plasma samples were deproteinized with Ba(OH)_2_ and ZnSO_4_, dried to remove ^3^H_2_O, resuspended in water, and counted in scintillation fluid (EcoLume) on channels for ^3^H and ^14^C. The plasma concentrations of ^3^H_2_O were determined by the difference between ^3^H counts without and with drying. For determination of tissue 2-deoxy-D-[1-^14^C]-glucose-6-phosphate (2-DG-6-P) content, tissue samples were homogenized and the supernatants were loaded to an ion-exchange column to separate 2-DG-6-P from 2-deoxy-D-[1-^14^C]-glucose.

## Results

### Clodronate liposomes reduce macrophage content in visceral adipose tissue of DIO mice

**Figure 1:**
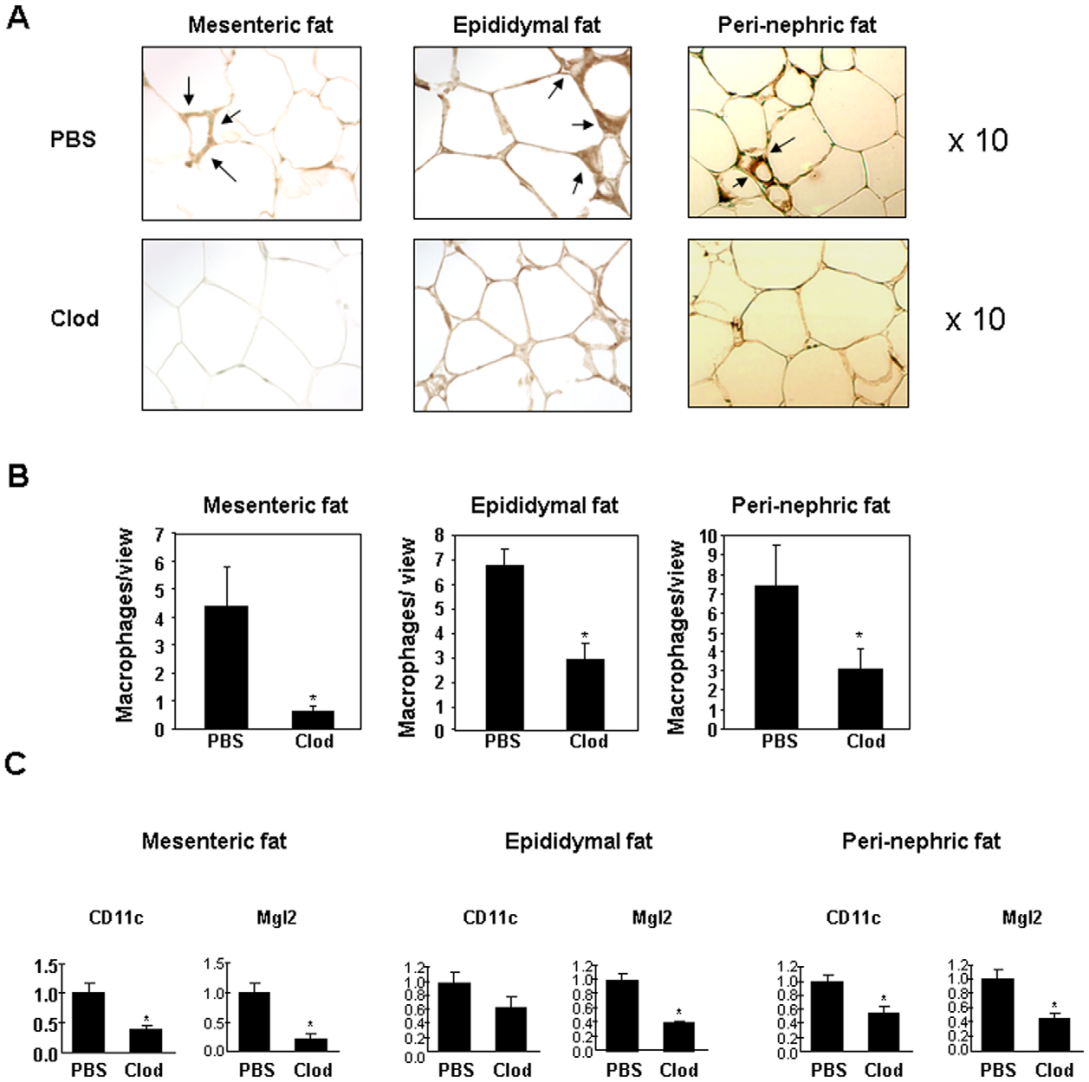
Effect of clodronate liposomes on macrophage depletion in fat of DIO mice. **A.** Histology images of adipose tissues from DIO mice. Mesenteric, epididymal and peri-renal fat pads from DIO mice treated with either PBS liposomes (PBS) or clodronate liposomes (Clod) by intraperitoneal injection were fixed, embedded, sectioned and stained with anti-F4/80 antibody (for mesenteric and epididymal fat pads, n = 9–13 per group; for peri-nephricl fat pads, n = 4 per group). Pictures were taken under 10 fold magnification. **B.** Quantification of adipose macrophages from DIO mice treated with PBS or clodronate liposomes. **C.** Expression of CD11c and Mgl2 in adipose tissues of DIO mice treated with either PBS liposomes or clodronate liposomes by intraperitoneal injection (n = 7 per group). * P<0.05, PBS liposomes treated vs. clodronate liposomes treated. In epididymal fat, the P value for CD11c between PBS and clod is 0.086.

To determine whether reduced visceral adipose macrophage content will be associated with a beneficial effect on improving insulin resistance in diet-induced obese mice, a chemical approach was used to deplete macrophages. To ablate adipose tissue macrophages, clodronate liposomes or PBS liposomes were injected twice into peritoneal cavities of lean or diet-induced obese mice with a three-day interval. Adipose tissue was harvested six days post the first injection for histological studies by performing immunohistochemistry with an anti-F4/80 antibody and for real-time PCR analysis for M1 and M2 macrophage marker genes. As shown in figure 1A–B, F4/80 positive cells are significantly decreased in adipose tissue from DIO mice treated with clodronate liposomes compared to control DIO mice injected with PBS liposomes. CD11c is a commonly accepted M1 marker gene although its expression is not exclusively restricted to macrophages. Expression levels of CD11c are decreased in visceral adipose depots (mesenteric, epididymal and peri-nephric) but not in subcutaneous adipose tissue (Figure 1C and figure S1A), indicating that peritoneal injection of clodronate liposomes selectively targeting visceral adipose depots without affecting subcutaneous fat. Expression levels of Mgl2, a M2 macrophage marker gene, are also reduced (Figure 1C and figure S1A). These data suggest that clodronate liposomes are non-selective regarding macrophage subtypes. Lean mice injected with clodronate liposomes also have reduced M1 and M2 macrophage content in visceral adipose depots (Figure S1B). The lean mice used in our study are age-matched controls for DIO mice and they are over twenty-four weeks old. Interestingly, adipose tissue of these lean mice has more proinflammatory macrophages compared to that of younger lean mice (Figure S2).

### Reduced visceral adipose macrophage content is associated with mildly improved systemic insulin sensitivity

**Figure 2:**
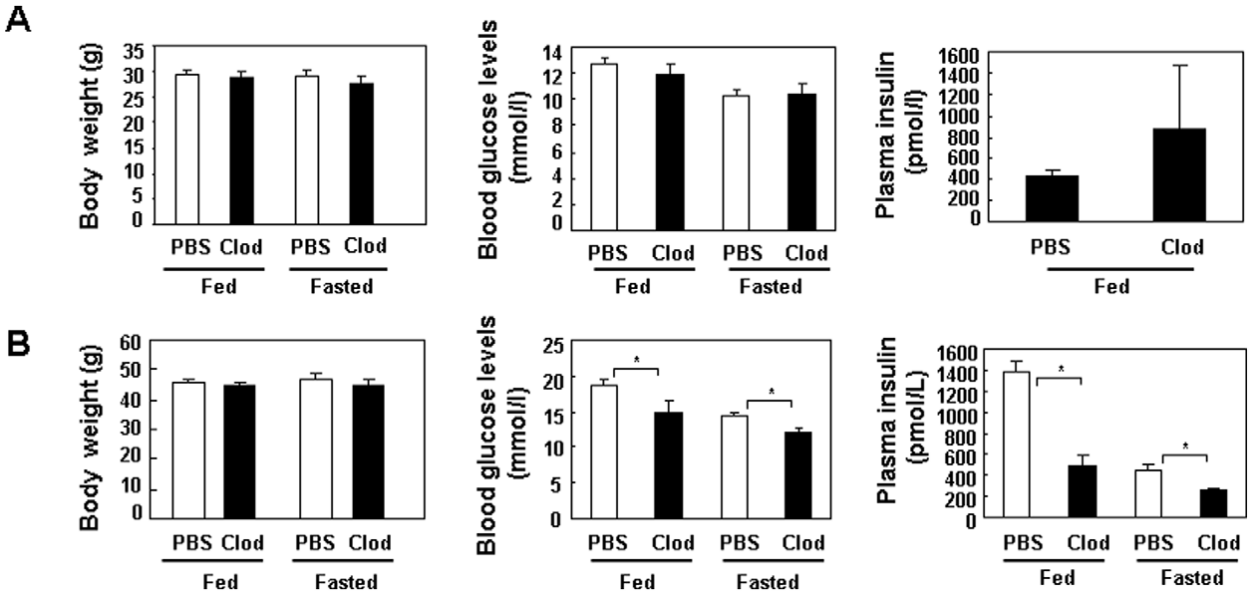
Body weight, blood glucose and plasma insulin levels in mice treated with clodronate liposomes or PBS liposomes. **A.** Body weight, blood glucose and plasma insulin levels of lean mice (n = 4–10 per group) 6–7 days post first injection. **B.** Body weight, blood glucose and plasma insulin levels of DIO mice (for BW and blood glucose measurement, n = 12–14 per group for fed condition; n = 7 per group for fasted condition; for insulin measurement, n = 5 per group for both fed and fasted conditions) 6–7 days post first injection. * P<0.05 clodronate liposomes treated vs. PBS liposome treated.

**Figure 3:**
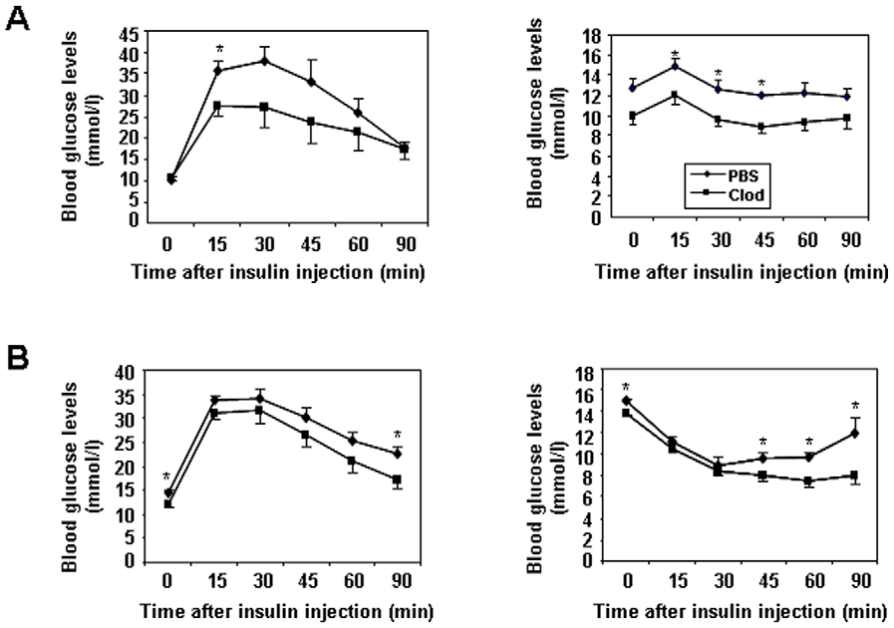
Glucose and insulin tolerance tests in mice treated with clodronate liposomes or PBS liposomes. **A.** Glucose and insulin tolerance tests in lean mice (n = 4–10 per group). **B.** Glucose and insulin tolerance tests in DIO mice (n = 7 per group). * P<0.05 clodronate liposomes treated vs. PBS liposome treated.

Injection of clodronate liposomes did not affect body weight of either lean or DIO mice under fed or fasted conditions (Figure 2). Postprandial or fasting blood glucose levels were not significantly altered in lean mice treated with clodronate liposomes vs. lean mice injected with PBS clodronate liposomes (Figure 2A). Postprandial plasma insulin levels in lean mice were not significantly altered either (Figure 2A). Postprandial and fasting blood glucose levels were significantly reduced in DIO mice treated with clodronate liposomes vs. control DIO mice treated with PBS liposomes (Figure 2B). Plasma insulin levels were also significantly reduced in DIO mice treated with clodronate liposomes compared to control DIO mice administered with PBS liposomes (Figure 2B). To test whether reduced visceral adipose macrophage content is associated with improved glycemic control, glucose and insulin tolerance tests were performed in both lean and DIO mice. Injection of clodronate liposomes mildly improved responses to glucose and insulin challenges in both lean and DIO mice (Figure 3).

### Clodronate liposomes improve hepatosteatosis in DIO mice

**Figure 4:**
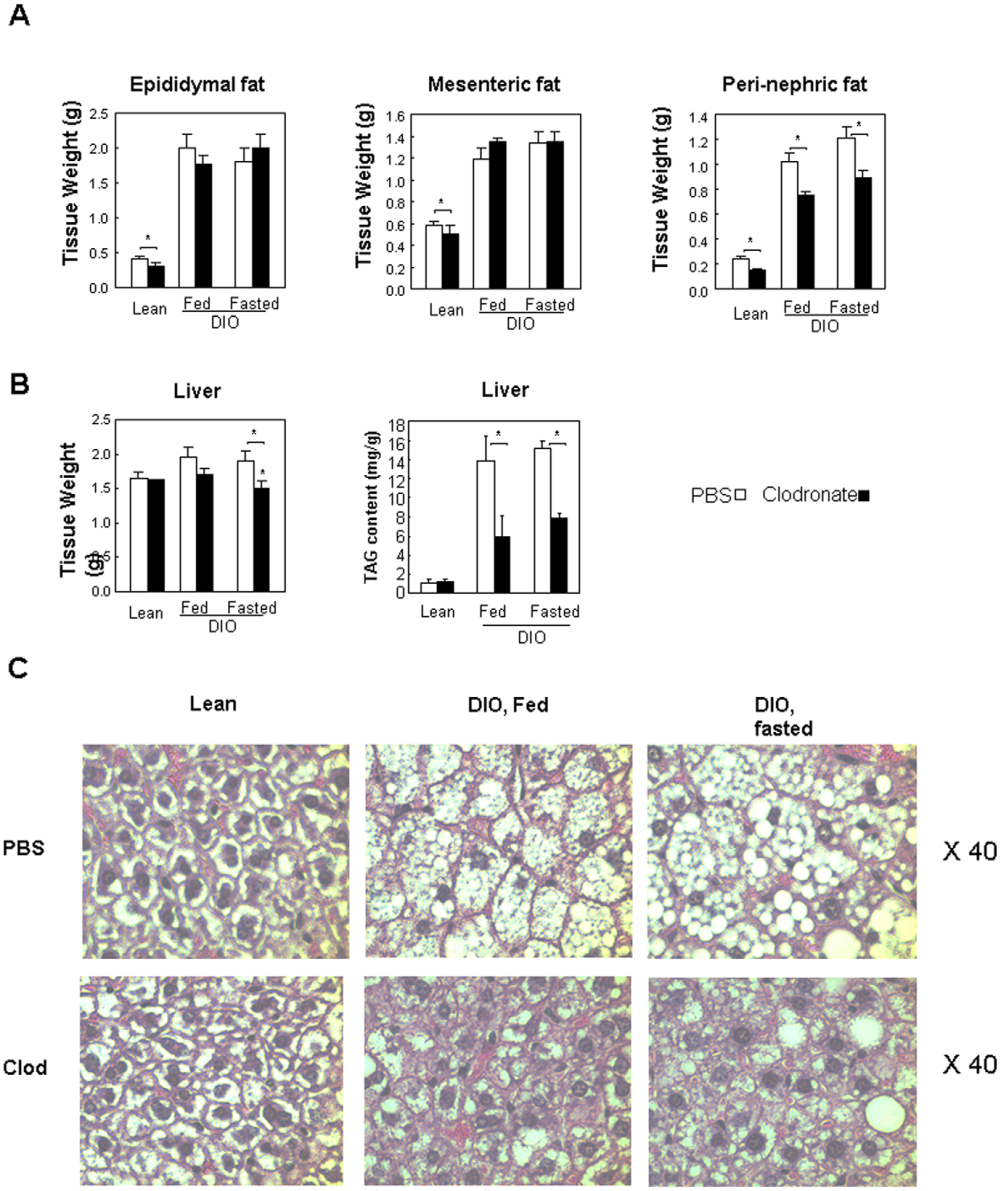
Tissue weights, TAG content and histology. **A.** Weights of adipose tissue. Epididymal, mesenteric and peri-nephric fat pads were harvested from lean or DIO mice injected with PBS liposomes or clodronate lipiosomes (For fed DIO mice, n = 6–7 per group; fasted DIO mice, n = 7 per group; lean mice, n = 3–5). **B.** Weights and triglyceride (TAG) contents of the livers from mice as described in A. **C.** Histology of the livers from mice as described in A. Liver section slides were stained with hematoxylin and eosin. Pictures were taken under 40 fold magnification. * P<0.05 clodronate liposomes treated vs. PBS liposomes treated.

**Figure 5:**
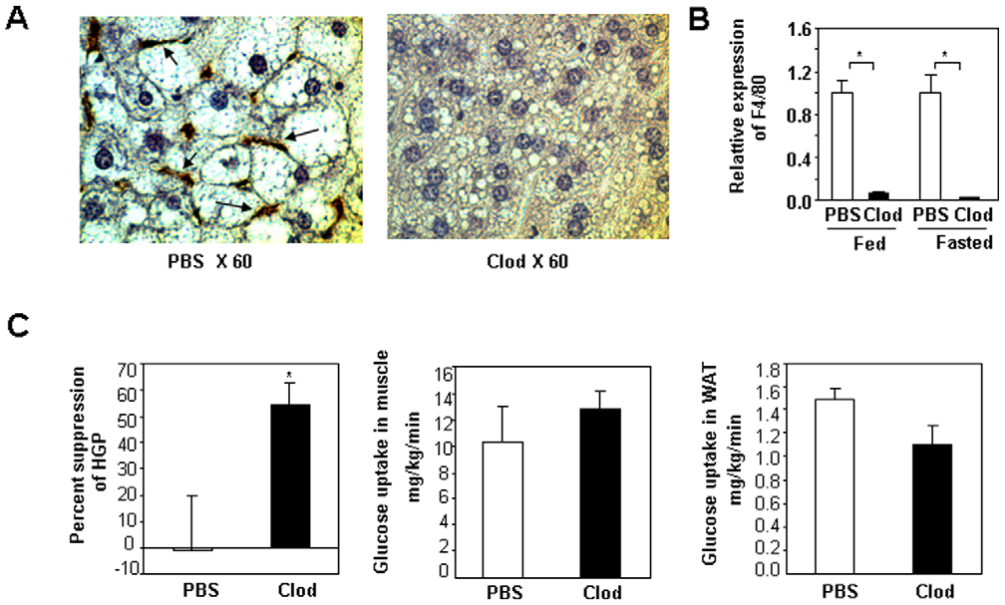
Effects of clodronate liposomes on liver. **A.** Deletion of kupffer cells in the liver of DIO mice by clodronate liposomes. Livers from DIO mice treated with either PBS liposomes (PBS) or clodronate liposomes (Clod) by intraperitoneal injection were fixed, embedded, sectioned and stained with anti-F4/80 antibody (n = 4 Per group). Pictures were taken under 60 fold magnification. **B.** Expression of F4/80 mRNA in the livers of DIO mice treated with either PBS liposomes or clodronate liposomes (n = 6–7 Per group). **C.** Hyperinsulinemic-euglycemic clamp study in DIOmice treated with clodronate liposomes or PBS liposomes (n = 4 per group). Percent suppression of hepatic glucose production (HGP), muscle and adipose glucose uptake were compared between DIO mice injected with clodronate liposomes or PBS liposomes. * P<0.05 clodronate liposomes treated vs. PBS liposomes treated. HGP, hepatic glucose production.

Dissection of mice revealed significant reduction of weights in epididymal, mesenteric and peri-nephric adipose tissue in lean mice injected with clodronate liposomes (Figure 4A). Significant reduction of weights in peri-nephric fat depot was observed in DIO mice injected with clodronate liposomes (Figure 4A). Liver weights were also significantly decreased in DIO mice injected with clodronate liposomes under fasted condition and trended lower under fed condition (Figure 4B). The reduction of liver weights is most likely due to decrease in triglyceride content (Figure 4B). Diet-induced obesity is associated with hepatosteatosis. To determine whether reduction in hepatic triglyceride content alleviated hepatotseatosis, histology studies were performed with livers from DIO mice. As shown in Figure 4C, massive amounts of lipid droplets were observed in the livers of DIO mice treated with PBS liposomes but were barely detectable in the livers of DIO mice treated with clodronate liposomes, indicating the absence of steatosis. Histology and real-time PCR studies showed depletion of macrophages in the livers of DIO mice treated with clodronate liposomes (Figure 5A–B). A hyperinsulinemic-euglycemic clamp study revealed significantly increased percent suppression of hepatic glucose output, suggesting that the ability of insulin on repressing glucose production in the liver is enhanced in DIO mice treated with clodronate liposomes compared to control DIO mice treated with PBS liposomes (Figure 5C). Glucose uptake in muscle and adipose tissue was not significantly altered in DIO mice treated with clodronate liposomes (Figure 5C). It has been reported that depletion of macrophages in visceral adipose tissue in lean mice by clodronate liposomes increases lipolysis and leads to elevated circulating FFA levels [33]. Increased FFA levels are known to be detrimental to systemic insulin sensitivity. Elevated plasma FFA levels are also observed in our DIO mice treated with clodronate liposomes (data not shown), suggesting that the beneficial phenotype of adipose macrophage reduction on improving systemic insulin sensitivity could be attenuated by increased lipolysis.

### Clodronate liposomes treatment affects profiles of cytokine and adipokine in circulation, adipocytes and stromal vascular cells

**Figure 6:**
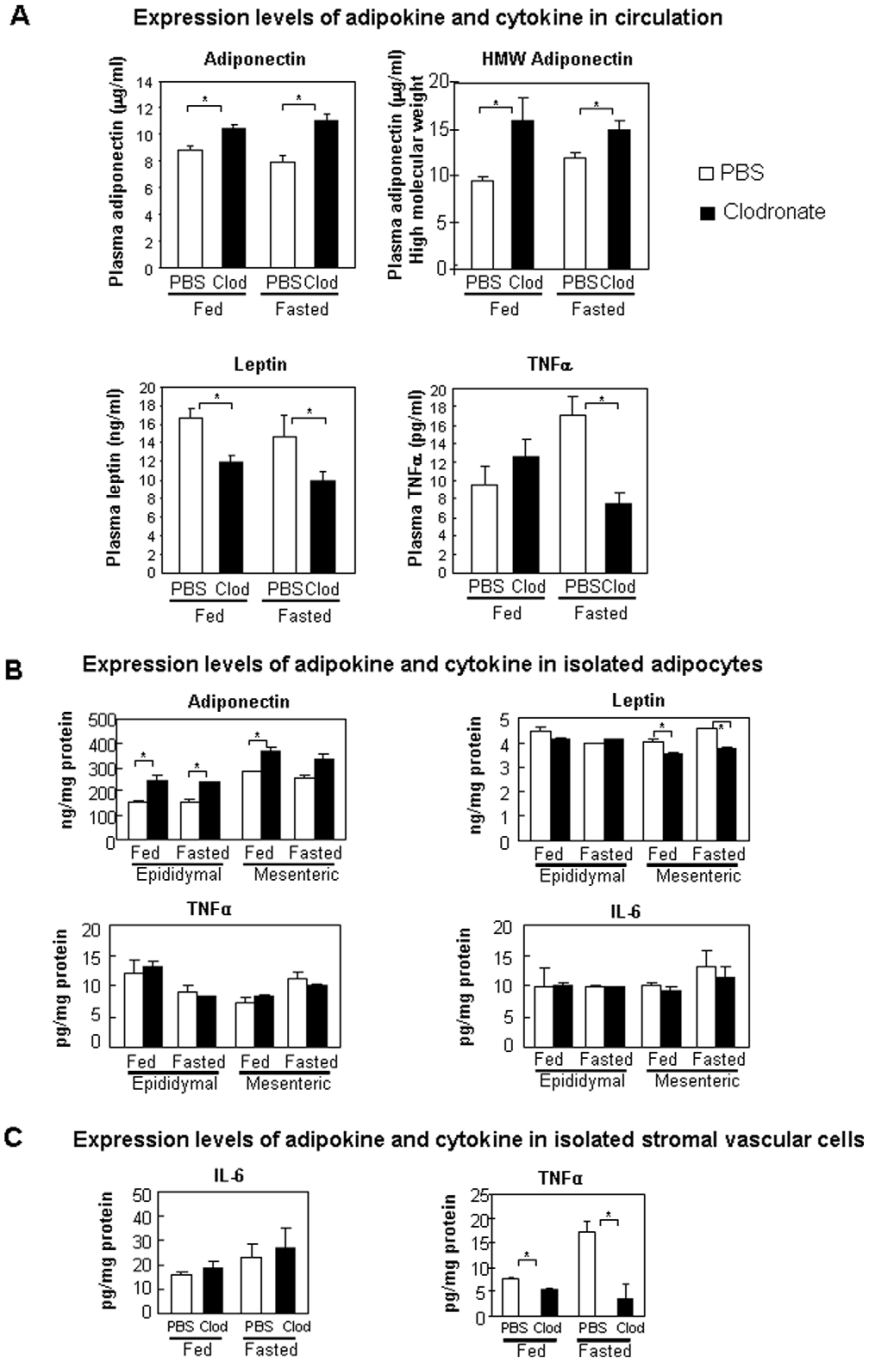
Effect of macrophage ablation on expression profiles of adipokine and cytokine. **A.** Expression levels of adipokine and cytokine in plasma from DIO mice ip injected with clodronate liposomes or PBS liposomes (Fed, n = 6–7 each group; fasted, n = 7 each group). **B.** Expression levels of adipokine and cytokine in isolated adipocytes from epididymal and mesenteric fat depots from DIO mice ip injected with clodronate liposomes or PBS liposomes. **C.** Expression levels of cytokine in stromal vascular cells isolated from epididymal fat depot of DIO mice ip injected with clodronate liposomes or PBS liposomes. * P<0.05, clodronate liposomes treated vs. PBS liposomes treated.

To explore the potential mechanism of improved insulin sensitivity by clodronate liposomes, expression levels of cytokine and adipokine were profiled in plasma, isolated primary adipocytes, and stromal vascular cells using ELISA. Circulating levels of adiponectin were significantly increased and leptin were significantly decreased in DIO mice injected with clodronate liposomes compared to control DIO mice treated with PBS liposomes (Figure 6A). Further analysis indicates that the functional high molecular weight form of adiponectin is also significantly increased (Figure 6A). Clodronate liposomes significantly reduced circulating levels of TNFα under fasting condition (Figure 6A) but surprisingly increased circulating levels of IL-6 (data not shown). Expression levels of adiponectin were also increased in isolated primary adipocytes harvested from epididymal and mesenteric fat depots of DIO mice injected with clodronate liposomes (Figure 6B). Leptin contents were not altered in adipocytes from epididymal fat depot but were significantly reduced in adipocytes from mesenteric fat depot of DIO mice treated with clodronate liposomes (Figure 6B). No change of IL-6 and TNFα content in adipocytes was observed in either fat depot (Figure 6B). In the stromal vascular fraction isolated from epididymal depot of DIO mice injected with clodronate liposomes, no change of IL-6 was observed and TNFα expression was significantly reduced (Figure 6C). Expression levels of TNFα were also significantly decreased in the liver of DIO mice treated with clodronate liposomes (Figure S3). The dramatic decrease of TNFα levels in both stromal cells of adipose tissue and livers of DIO mice can potentially contribute to lowered fasting TNFα levels in circulation.

### Macrophage-adipocyte co-culture reduces adiponectin expression

**Figure 7:**
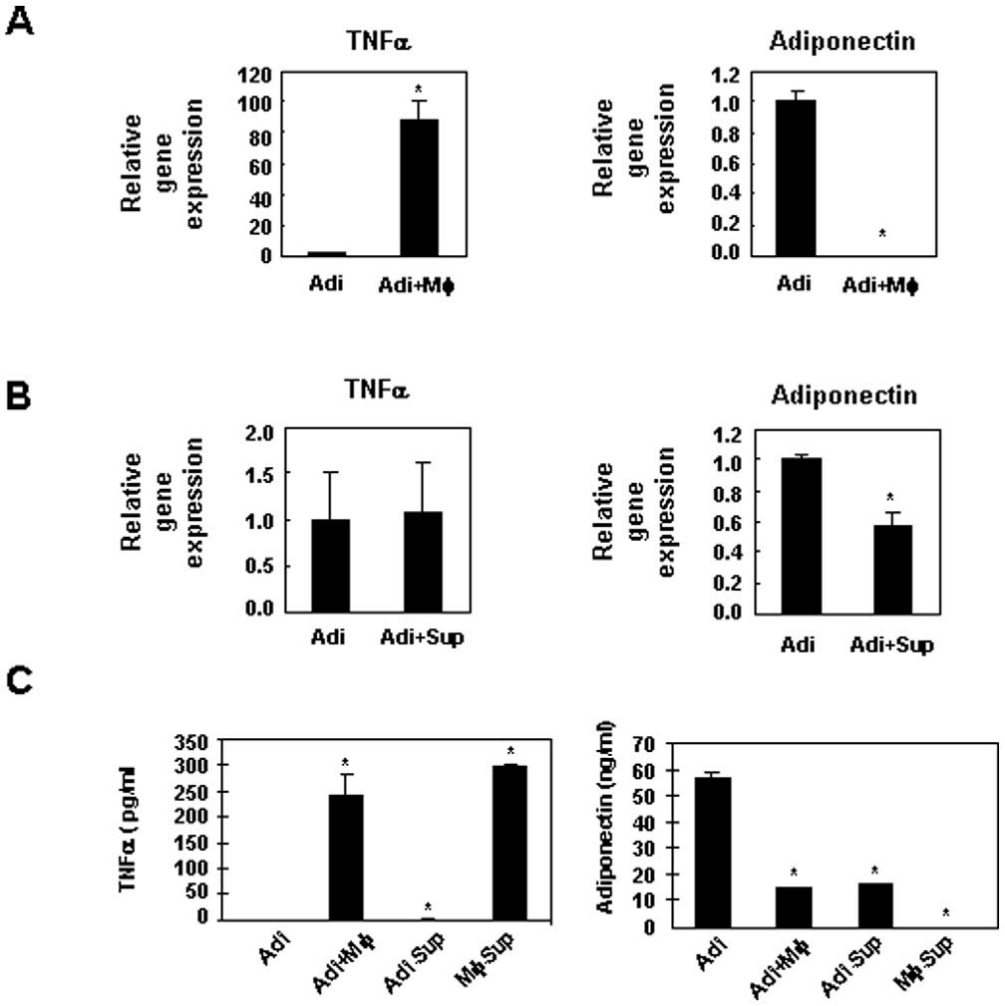
3T3-L1 adipocytes and Raw264.7 macrophage co-culture experiment. **A.** Expression of TNFα and adiponectin in L1 adipocytes (Adi) and L1+macrophages (Mφ). Macrophages were seeded on top of fully differentiated adipocytes for two days, then co-cultured cells were incubated in serum-free medium overnight prior to RNA extraction. **B.** Expression of TNFα and adiponectin in L1 adipocytes treated with normal medium or conditioned medium from macrophages (Sup). **C.** Expression of TNFα and adiponectin in conditioned media from adipocytes (Adi), adipocyte and macrophage co-culture (Adi+ Mφ), adipocyte twenty four hours after removal of conditioned macrophage supernatant, or macrophage supernatant. *P<0.05 compared to adipocyte control. Data shown are representative experiment out of three independent experiments performed.

Adipocyte is an important source of adiponectin. To investigate the mechanism of increased adiponectin expression upon injection of clodronate liposomes, we co-cultured 3T3-L1 murine adipocytes and non-stimulated Raw264.7 macrophage line, the latter produces a large amount of TNFα under b asal condition. We also treated 3T3-L1 adipocytes with conditioned media derived from Raw264.7 macrophages. The expression level of adiponectin in cultured adipocytes was significantly reduced by co-culture with macrophages or upon exposure to conditioned media from macrophages (Figure 7), suggesting that macrophage secreted factors, possibly TNFα, are responsible for repressing adiponectin production. Increased adiponectin levels in DIO mice injected with clodronate liposomes are most likely due to adipose macrophage depletion.

## Discussion

Macrophage infiltration, accumulation and activation in adipose tissue of obese animals have been considered to play an important role in the development of obesity-related insulin resistance and type 2 diabetes. Visceral adipose tissue is the fat depot correlated with obesity-related metabolic syndrome and reduction of macrophage content in visceral adipose tissue may be beneficial for improving metabolic syndrome in obese animals. Despite the fact that a genetic approach was used previously to deplete CD11c positive macrophages in adipose tissue and improved systemic insulin sensitivity was observed [22], this approach theoretically also eliminates macrophages in subcutaneous adipose tissue and any tissue that contains macrophages. In addition, this approach is also expected to deplete non-macrophages [26]. In our study, we used a chemical reagent to locally ablate macrophages in visceral adipose tissue. Injection of clodronate liposomes into peritoneal cavity of mice successfully reduced macrophage contents in fat depots located within peritoneal cavity, particularly effective in mesenteric adipose tissue. Due to the fact that clodronate liposomes can not penetrate vascular barriers, macrophage content in subcutaneous adipose tissue is not altered. This approach mildly improved glucose and insulin tolerance in both lean and diet induced obese mice without affecting body weight. These results support the hypothesis that visceral adipose macrophage accumulation at least partially contributes to the development of obesity-related insulin resistance and hyperglycemia. The beneficial phenotype is mild, which could be due to undesired complication of M2 macrophage depletion and elevated circulating FFA levels, both are detrimental to systemic insulin sensitivity.

Livers are also readily accessible by clodronate liposomes injected into peritoneal cavity. Three studies reported deletion of liver kupffer cells by clodronate liposomes in diet-induced mice [29]–[31]. These studies applied a much lower (or an unspecified) dose of clodronate liposomes. The authors did not observe a beneficial effect on systemic glucose homeostasis and neither did they report any effect on adipose tissue macrophage ablation. Despite successful deletion of kupffer cells in all studies, contradictory conclusions regarding the role of kupffer cells in obesity-related hepatosteatosis and hepatic insulin resistance are made. In our study, dramatic reduction of triglyceride content was observed in the livers of DIO mice treated with clodronate liposomes which essentially eliminated obesity-associated hepatosteatosis. We previously reported that obesity-induced inflammation was specifically located to adipose tissue, not liver or other organs although there are residential macrophages in the liver [6]. Therefore, we speculate that improved hepatosteatosis may be a secondary effect of macrophage reduction in visceral adipose tissue. Indeed, depletion of kupffer cells in DIO mice by intravenous injection of clodronate liposomes, which did not affect visceral adipose macrophage content, did not alter liver triglyceride content, had no improvement on hepatosteatosis or hyperglycemia (data not shown). Therefore macrophage reduction in other tissue/organ other than liver itself may account for attenuated hepatosteatosis and improved glucose/insulin tolerance. One possibility could be that alteration of macrophage content in visceral adipose tissue may cause changes of secreted factors that can potentially impact liver.

Expression profiles of adipokines and cytokines in diet induced mice were profoundly altered by administration of clodronate liposomes. Adiponection is an anti-inflammatory and insulin sensitizing hormone [34]. Circulating levels of adiponectin are negatively correlated with body fat and are decreased in obese and diabetic patients. Treatment with clodronate liposomes increased adiponectin levels in both adipocytes and circulation. It is possible that elevated circulating levels of adiponectin contributed to the improvement of hepatosteatosis in DIO mice treated with clodronate liposomes. It has been reported that adiponectin deficient mice develop hepatosteatosis whereas elevation of adiponectin levels in circulation through pharmacological and genetic approaches protect mice from developing obesity-related hepatosteatosis [35], [36]. Adiponectin is also considered a good marker for predicting severity of non-alcoholic fatty liver disease and recent therapeutic approaches have been focused on indirectly increasing adiponectin levels [37]. The potential mechanisms by which adiponectin improves hepatosteatosis include involvement of multiple pathways such as activation of SIRT1 and AMP-activated protein kinase, promotion of fatty acid oxidation in the liver, and suppression of toll-like receptor 4 signaling [38], [39]. Future studies will be necessary to explore whether adiponectin alleviates hepatosteatosis in DIO mice injected with clodronate liposomes via aforementioned pathways. Leptin, on the other hand, is a pro-inflammatory hormone that is positively correlated with body fat and is elevated in circulation of obese and diabetic patients [34]. Leptin levels in circulation and mesenteric adipocytes were decreased by treatment of clodronate liposomes. TNFα expression levels were decreased in stromal cells of adipose tissue and liver of DIO mice injected with clodronate liposomes, which may contribute to lowered circulating TNFα levels under fasting condition. Decreased TNFα level possibly contributes to increased adiponectin level as substantial evidence in literature demonstrate that TNFα inhibits adiponectin expression [40]–[44]. Our study also indicates that TNFα-producing macrophages can decrease adiponectin expression and release in 3T3-L1 adipocytes during co-culture. Alteration of circulating adipokine and cytokine levels provides a possible explanation that locally administered clodronate liposomes were capable of mildly improving systemic insulin sensitivity. We do not exclude the possibilities that macrophage reduction in other organs located in the peritoneal cavity (such as spleen, intestine etc) also contribute to improved systemic insulin sensitivity. In summary, it is feasible to improve metabolic profile of DIO mice using the chemical approach of macrophage ablation. Chemical reagents selectively ablate M1 macrophages may have a bigger beneficial effect.

## Supporting Information

Figure S1 **Macrophage contents in adipose tissue from DIO and lean mice injected with clodronate liposomes. A.** Expression levels of CD11c and Mgl2 in subcutaneous adipose tissue of DIO mice treated with clodronate or PBS liposomes by intraperitoneal injection (n = 5 per group). **B.** Expression levels of CD11c and Mgl2 in adipose tissues from lean mice treated with clodronate or PBS liposomes by intraperitoneal injection (n = 4–5 per group). * P<0.05, PBS vs. clodronate liposomes. Clod, clodronate liposomes.
(TIF)

Figure S2 **Expression levels of CD11c in epididymal adipose tissue of five and twenty-five week-old leanC57BL/6 male mice (n = 5–7).** * P<0.05, 5 week vs. 25 week old mice.
(TIF)

Figure S3 **TNFα expression in liver from DIO mice treated with PBS or clodronate liposomes (n = 6–7 per group).** Livers from DIO mice treated with PBS liposomes (PBS) or clodronate liposomes (Clod) by IP injection were harvested and used for RNA preparation. TNFα expression was determined by real-time PCR analysis. *P<0.05, PBS vs. clodronate liposomes.
(TIF)
