# Supplementary figures and images for: Clodronate Liposomes Improve Metabolic Profile and Reduce Visceral Adipose Macrophage Content in Diet-Induced Obese Mice
Source: PLoS One. 2011 Sep 12;6(9):e24358. doi: 10.1371/journal.pone.0024358 (PMC3171445; doi:10.1371/journal.pone.0024358)

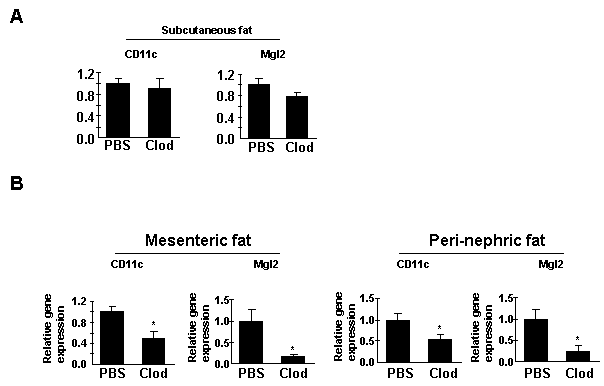

Supplement: Figure S1 — Macrophage contents in adipose tissue from DIO and lean mice injected with clodronate liposomes. A. Expression levels of CD11c and Mgl2 in subcutaneous adipose tissue of DIO mice treated with clodronate or PBS liposomes by intraperitoneal injection (n = 5 per group). B. Expression levels of CD11c and Mgl2 in adipose tissues from lean mice treated with clodronate or PBS liposomes by intraperitoneal injection (n = 4–5 per group). * P<0.05, PBS vs. clodronate liposomes. Clod, clodronate liposomes. (TIF) [file pone.0024358.s001.tif]

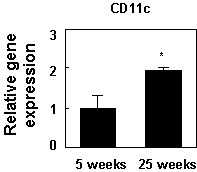

Supplement: Figure S2 — Expression levels of CD11c in epididymal adipose tissue of five and twenty-five week-old leanC57BL/6 male mice (n = 5–7). * P<0.05, 5 week vs. 25 week old mice. (TIF) [file pone.0024358.s002.tif]

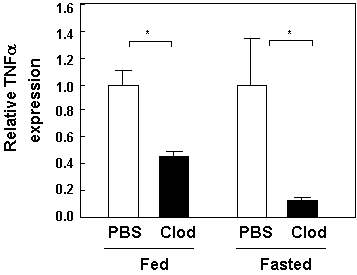

Supplement: Figure S3 — TNFα expression in liver from DIO mice treated with PBS or clodronate liposomes (n = 6–7 per group). Livers from DIO mice treated with PBS liposomes (PBS) or clodronate liposomes (Clod) by IP injection were harvested and used for RNA preparation. TNFα expression was determined by real-time PCR analysis. *P<0.05, PBS vs. clodronate liposomes. (TIF) [file pone.0024358.s003.tif]
